# Case of resected multiple hepatocellular adenomas in a young man with severe obesity

**DOI:** 10.1186/s40792-019-0689-3

**Published:** 2019-08-13

**Authors:** Kentaro Oji, Takeshi Urade, Yoshiteru Iwatani, Katsuhide Tanaka, Hirotaka Hirano, Tsuyoshi Sanuki, Masaru Tomita, Yuki Yamamoto, Yoh Zen, Daisuke Kuroda

**Affiliations:** 1 Department of Surgery and Digestive Surgery, Kita-Harima Medical Center, 926-250, Ichiba-cho, Ono, 675-1392 Japan; 2 Department of Gastroenterology, Kita-Harima Medical Center, 926-250, Ichiba-cho, Ono, 675-1392 Japan; 3 Division of Gastroenterology, Department of Internal Medicine, Kobe University Graduate School of Medicine, 7-5-2, Kusunoki-cho, Chuo-ku, Kobe, 650-0017 Japan; 4 Department of Medical Radiography, Kita-Harima Medical Center, 926-250, Ichiba-cho, Ono, 675-1392 Japan; 5 Department of Diagnostic Pathology, Kita-Harima Medical Center, 926-250, Ichiba-cho, Ono, 675-1392 Japan; 6 Department of Diagnostic Pathology, Kobe University Graduate School of Medicine, 7-5-2, Kusunoki-cho, Chuo-ku, Kobe, 650-0017 Japan; 7 IHU Strasbourg Institute of Image-Guided Surgery, 1, Place de l’Hôpital, 67091 Strasbourg, CEDEX France

**Keywords:** Hepatocellular adenoma, Hepatocellular adenomas, Laparoscopic hepatectomy, Laparoscopic liver resection, Liver cell adenoma, Obesity

## Abstract

**Background:**

Hepatocellular adenoma (HCA) is a rare liver tumor that has the potential for rupture and malignant transformation. Here, we report a case of multiple hepatocellular adenomas (HCAs) that were treated by surgical resection.

**Case presentation:**

An 18-year-old man was admitted to our hospital with proteinuria. His height was 176.5 cm, weight was 126 kg, and body mass index was 40 kg/m^2^. A liver tumor was incidentally found on abdominal ultrasonography. Contrast-enhanced computed tomography and gadoxetic acid-enhanced magnetic resonance imaging revealed three hepatic tumors that were 68 mm, 16 mm, and 9 mm in segments 3/4, 8, and 1, respectively. A percutaneous needle biopsy of the largest tumor was performed, the diagnosis of unclassified type HCA was made, and laparoscopic partial liver resection was performed of all three. The postoperative course was uneventful, and the patient was discharged 12 days later. An immunohistochemical examination revealed positivity for serum amyloid A protein, no decrease in fatty acid-binding protein, and negativity for β-catenin, glutamine synthetase, and cytokeratin 7. Therefore, these tumors were diagnosed as inflammatory type HCAs.

**Conclusions:**

We reported an extremely rare case of multiple resected HCAs in a young, obese Japanese man. Our findings suggest that HCA should be considered in the differential diagnosis of liver tumor in obese patients. Further studies that consider clinical and molecular risk factors are required to establish individualized treatment plans for HCA in obese patients.

## Background

Hepatocellular adenoma (HCA) is a rare benign liver tumor, most frequently affecting young women with a history of oral contraceptive use [1]. HCA is rare in children, men, and the elderly; 85% of cases affect young women [2]. The estimated annual HCA incidence is 3 per 1,000,000 people but might be 10 times higher in long-term users of high-dose oral contraceptives in Europe and North America [1, 3]. HCA is extremely rare in Asian countries including Japan, where oral contraceptives are used far less often than in Western countries [4]. Before 2015, only 63 HCA cases were reported in a nationwide survey in Japan [5]. Other risk factors for HCA include anabolic steroid exposure and rare pathological conditions including Fanconi anemia, glycogen storage disease, and familial adenomatous polyposis [6]. Obesity and metabolic syndrome are also increasingly recognized as risk factors [7, 8].

Here, we report a rare case of multiple hepatocellular adenomas (HCAs) in a young Japanese man with severe obesity that was treated with laparoscopic liver resection (LLR).

## Case presentation

**Fig. 1:**
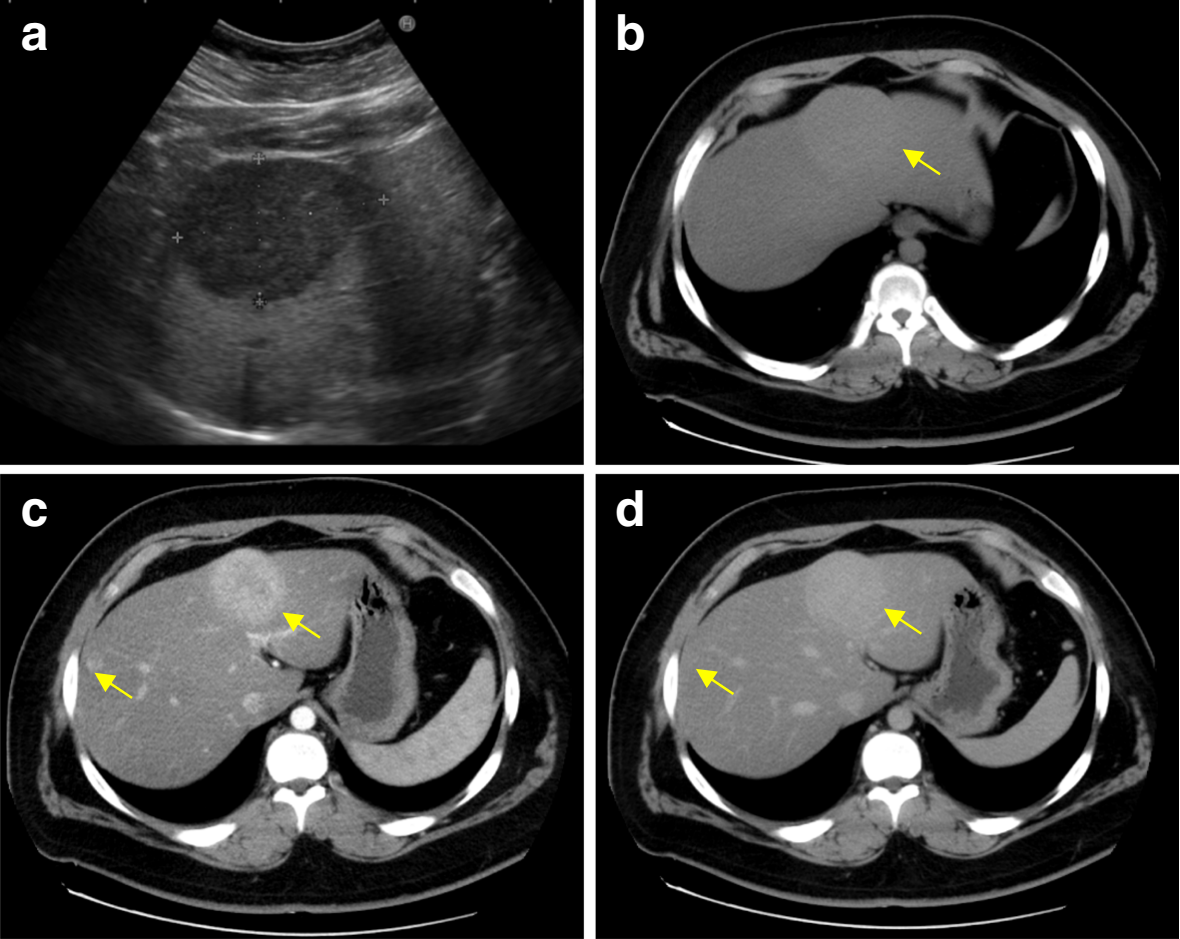
Preoperative ultrasonography and computed tomography images showing the presence of tumors. **a** Abdominal ultrasonography image showing a hypoechoic tumor approximately 7 cm in diameter protruding forward on the surface of the left lobe. **b** Plain computed tomography image showing an isodense tumor in the left lobe. **c** Contrast-enhanced computed tomography (CECT) image showing two tumors in segments 3/4 and 8 that were enhanced in the arterial phase. **d** CECT showing the two tumors with prolonged enhancement in the late phase

**Fig. 2:**
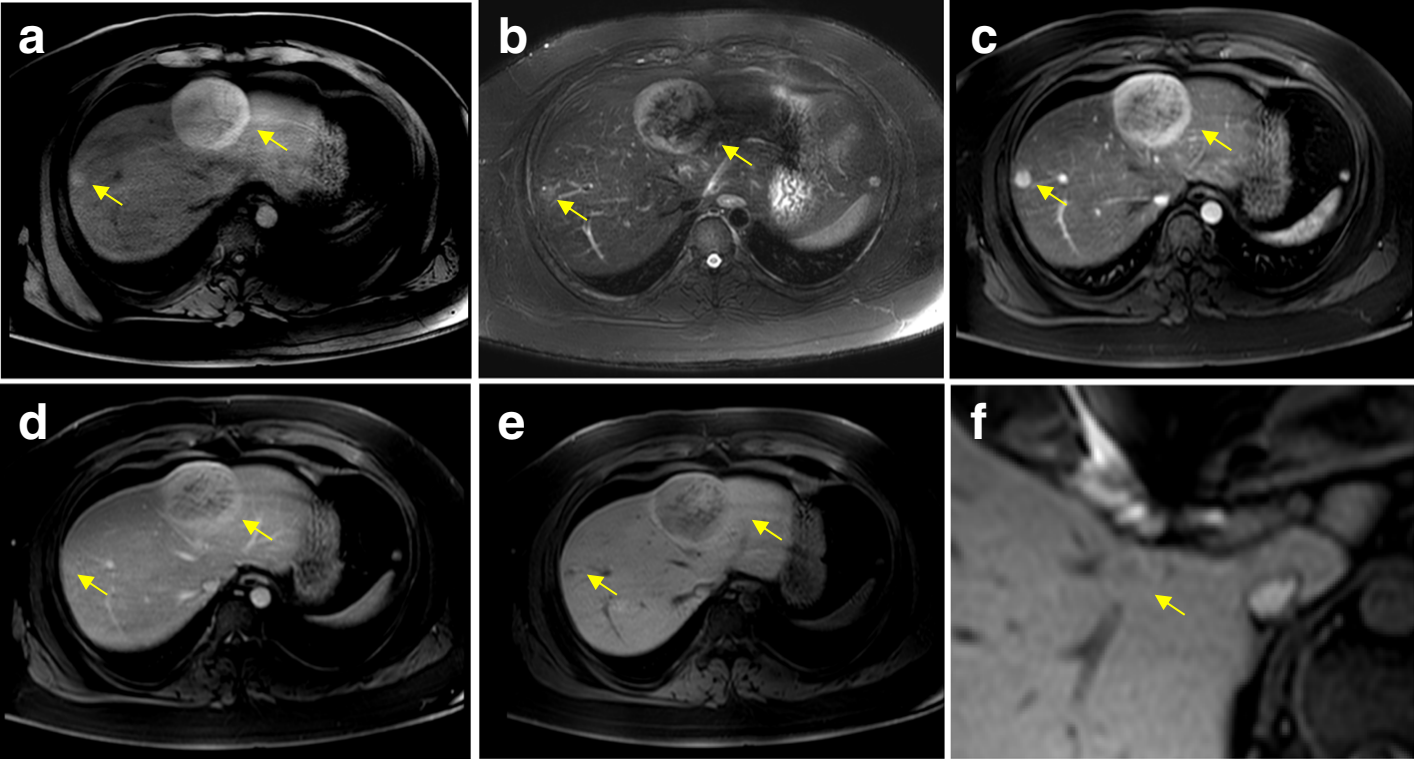
Preoperative magnetic resonance imaging showing the presence of tumors. **a** T1-weighted magnetic resonance image (MRI) showing the two hyperintense tumors. **b** T2-weighted MRI showing the two tumors with mild hypertensity. **c** Gadoxetic acid-enhanced (EOB)-MRI showing the two tumors with enhancement in the arterial phase. **d** EOB-MRI showing the two tumors with prolonged partial enhancement in the late phase. **e** EOB-MRI showing the two tumors in segments 3/4 and 8 with low signal intensity in the hepatocellular phase. **f** EOB-MRI showing the one tumor in segment 1 with low signal intensity in the hepatocellular phase

**Fig. 3:**
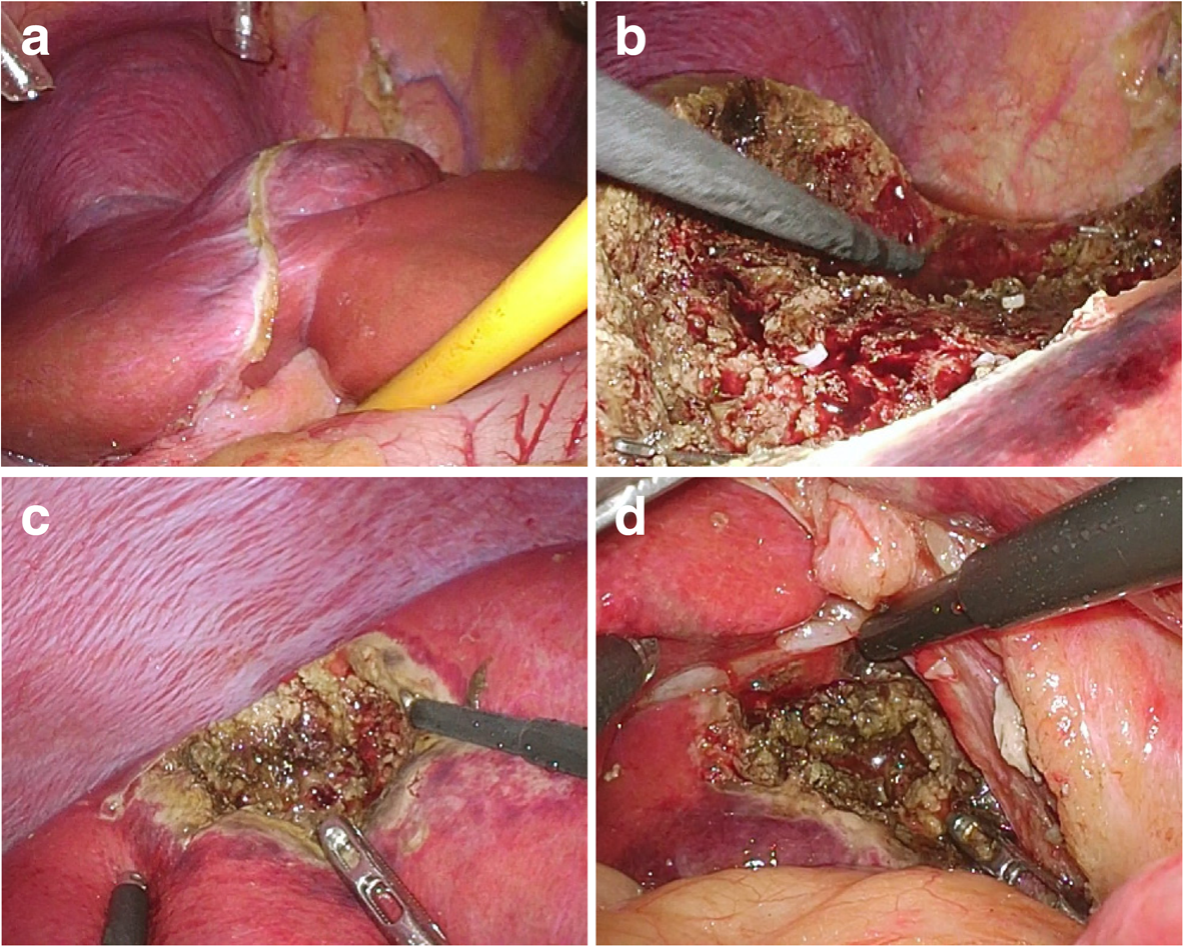
Intraoperative findings during the laparoscopic partial liver resection. **a** Laparoscopy showing the largest tumor, which protruded out of the left lobe. **b** Gross appearance of the cut surface of segment 3/4. **c** Gross appearance of the cut surface of segment 8. **d** Gross appearance of the cut surface of segment 1

**Fig. 4:**
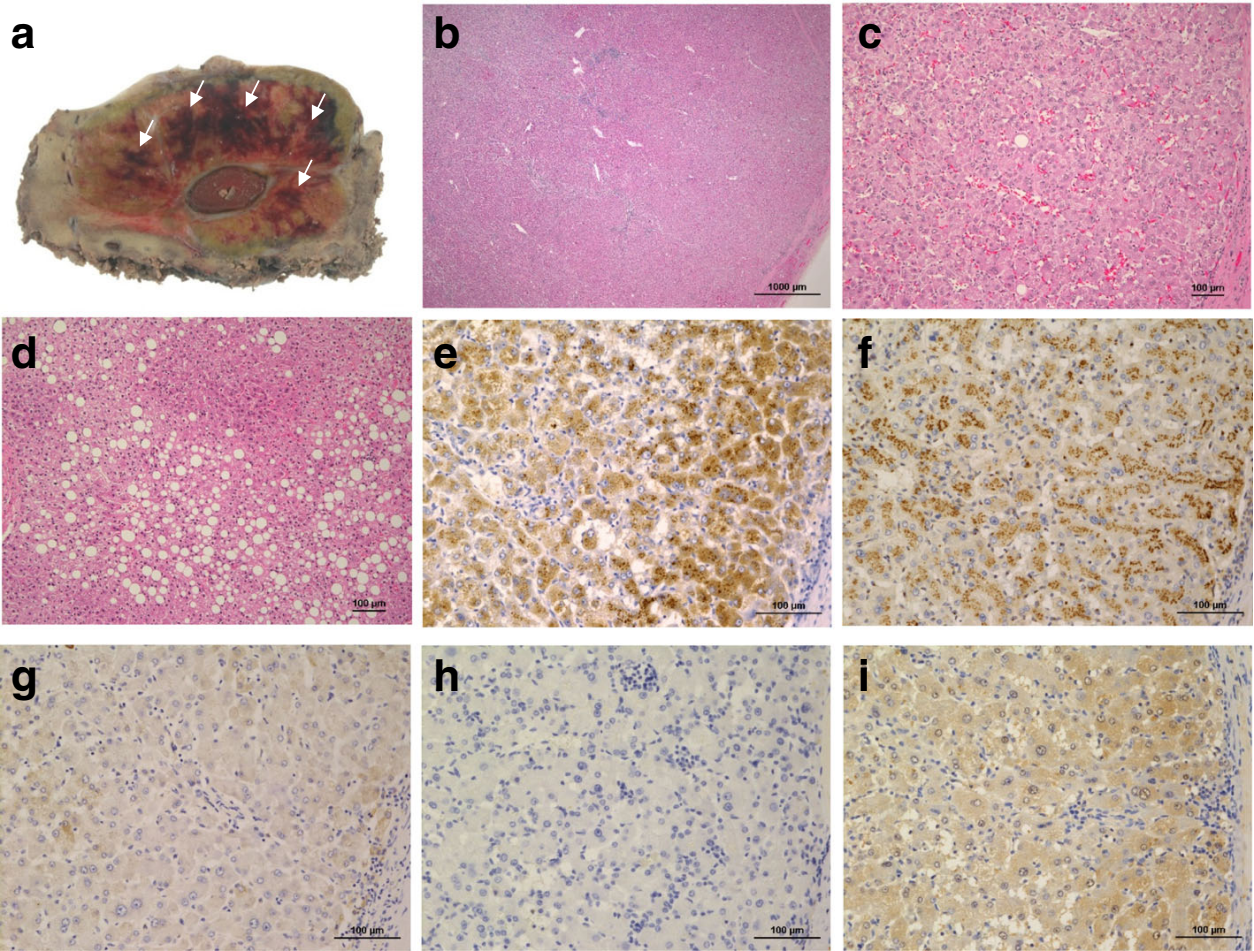
Histopathological findings of the resected specimen. **a** Macroscopically, the tumors were yellowish and well-defined with hemorrhage (white arrows). **b**, **c** Pathological examination revealed slightly enlarged hepatocyte proliferations without nuclear atypia or mitosis. (Hematoxylin and eosin [H&E] staining, original magnification: **b** H&E, × 20; **c** H&E, × 100) **d** The background liver was steatotic with mild fibrosis (H&E, × 100). **e**–**i** Immunohistochemical examination revealed the following findings of the tumor cells (immunohistochemical staining, × 200): positive for serum amyloid A protein (**e**), negative for β-catenin (**f**), negative for glutamine synthetase (**g**), negative for cytokeratin 7 (**h**), and positive for fatty acid-binding protein (**i**)

An 18-year-old man was admitted to our hospital with proteinuria identified in a medical checkup. He had no personal or familial medical history. His height was 176.5 cm, weight was 126 kg, and body mass index (BMI) was 40 kg/m^2^. He was taking no medication and had never consumed alcohol. A physical examination demonstrated no findings. Laboratory investigation results are shown in Table 1. Hepatic dysfunction, dyslipidemia, and slight increases in C-reactive protein were noted in the laboratory findings. All hepatitis viral markers were negative. An elevated serum protein level induced by vitamin K absence or antagonist-II (PIVKA-II) level of 92 mAU/mL was observed. Abdominal ultrasonography showed a liver tumor approximately 7 cm in diameter protruding forward with a hypoechoic and smooth surface on the left lobe (Fig. 1a). Abdominal plain computed tomography showed an isodense tumor in the left lobe (Fig. 1b). Contrast-enhanced computed tomography (CECT) showed two tumors in Couinaud’s segments 3/4 and 8 (Fig. 1c, d) that showed enhancement in the early phase and prolonged enhancement in the late phase. The two tumors expressed hyperintensity on T1-weighted magnetic resonance imaging (MRI) and mild hyperintensity on T2-weighted MRI (Fig. 2a, b). Gadoxetic acid-enhanced (EOB)-MRI showed that the two tumors had similar contrast attitudes on CECT in the early and late phases (Fig. 2c, d). EOB-MRI also revealed three tumors with low signal intensity in the hepatocellular phase (Fig. 2e, f) that were 68 mm, 16 mm, and 9 mm in size in segments 3/4, 8, and 1, respectively. They were suspected as either HCAs, focal nodular hyperplasia, lymphoma, or hepatocellular carcinoma (HCC) based on the imaging findings. A percutaneous needle biopsy was performed on the largest tumor. Microscopic pathological examination revealed that the tumor had slightly larger hepatocyte proliferation, expanded muscle-type arteries, scattered sinusoids, and inflammatory cell invasion but no nuclear atypia. An immunohistochemical examination revealed that the tumor cells were negative for serum amyloid A protein and β-catenin and partially positive for glutamine synthetase. Downregulation of liver fatty acid-binding protein was not observed. The cells also tested negative for cytokeratin 7 and MIB1. Accordingly, the tumor was diagnosed as an unclassified type HCA. Although the patient attempted to lose weight with diet and exercise before surgery, his weight had not decreased and the tumor size was unchanged 6 months after the diagnosis. The patient was scheduled to undergo laparoscopic partial liver resection for the largest HCA, which was larger than 5 cm and carried potential risks of rupture and malignant transformation. Finally, laparoscopic partial liver resection was performed on all three HCAs as we found using ultrasound that the other two small tumors were located near the liver surface. The intraoperative findings are shown in Fig. 3a–d. The operative time was 397 min, and a blood loss of 32 mL. A blood transfusion was not needed. A gross pathological examination revealed that the tumors were yellowish, well-defined, and 57 × 45 mm, 9 mm, and 5 mm in size, in segments 3/4, 1, and 8, respectively (Fig. 4a). The largest HCA had a hemorrhage. A pathological examination revealed slightly larger hepatocyte proliferation, expanded muscle-type arteries, and scattered sinusoids within the tumors (Fig. 4b, c). It also revealed a hematoma with hemosiderosis in the red areas of the tumors. The background of the liver was steatotic (70%) with mild fibrosis (Fig. 4d). Immunohistochemistry revealed that the tumor cells were positive for serum amyloid A protein, negative for β-catenin, negative for glutamine synthetase, and negative for cytokeratin 7 without downregulation of fatty acid-binding protein (Fig. 4e–i). Based on the findings, all the tumors were diagnosed as inflammatory type HCAs. The postoperative course was uneventful, and the patient was discharged 12 days after the procedure. The serum PIVKA-II decreased to 27 mAU/mL 8 months after discharge. As of 21 months postoperative follow-up, no recurrence was noted.

**Table 1:** Patient’s laboratory data at admission

Laboratory test	Value	Normal range
White blood cells	7100/μL	4000–8500/μL
Red blood cells	583 × 10^4^/μL	4.15–5.50 × 10^4^/μL
Hemoglobin	16.5 g/dL	13.5–17.5 g/dL
Platelets	24.1 × 10^4^/μL	12–36 × 10^4^/μL
Prothrombin time	88.7%	80–125%
Sodium	140.9 mEq/L	136–147 mEq/L
Potassium	4.01 mEq/L	3.5–5.0 mEq/L
Chloride	102.5 mEq/L	98–108 mEq/L
Total protein	8.0 g/dL	6.5–8.2 g/dL
Albumin	4.5 g/dL	3.8–5.3 g/dL
Total bilirubin	1.00 mg/dL	0.3–1.2 mg/dL
Aspartate aminotransferase	38 U/L	8–40 U/L
Alanine aminotransferase	105 U/L	5–45 U/L
Alkaline phosphatase	475 U/L	100–340 U/L
Gamma-glutamyl transpeptidase	127 U/L	0–75 U/L
Lactate dehydrogenase	205 U/L	115–245 U/L
Cholinesterase	467 U/L	239–485 U/L
Total cholesterol	254 mg/dL	130–219 mg/dL
High-density lipoprotein cholesterol	49 mg/dL	40–85 mg/dL
Low-density lipoprotein cholesterol	168 mg/dL	70–139 mg/dL
Triglyceride	212 mg/dL	30–149 mg/dL
Blood urea nitrogen	11.5 mg/dL	8.0–23.0 mg/dL
Creatinine	0.70 mg/dL	0.61–1.08 mg/dL
C-reactive protein	1.32 mg/dL	0–0.30 mg/dL
Hemoglobin A1c	6.2%	< 6.0%
Hepatatis B surface antigen	Negative	Negative
Hepatitis C virus antibody	Negative	Negative
Anti-nuclear antibody	< × 40	< × 40
Anti-mitochondrial M2 antibody	< 1.5 INDEX	0–6.99 INDEX
Alfa-fetoprotein	< 2.00 ng/mL	0–10 ng/mL
Carcinoembryonic antigen	1.8 ng/mL	0–5 ng/mL
Carbohydrate antigen 19-9	4.58 U/mL	0–2 U/mL
Protein induced by vitamin K absence or antagonist-II	92 mAU/mL	< 40 mAU/mL
Neuron-specific enolase	10.4 ng/mL	< 16.3 ng/mL
Soluble interleukin-2 receptor	397 U/mL	145–519 U/mL
Indocyanine green 15-min retention rate	15.0%	< 10%

## Discussion

HCAs are rare in children, men, and the elderly. The male-to-female ratio in the incidence of HCA is approximately 1:10 [9]. The dominant risk factor of HCA is oral contraceptive use. Exposure to estrogen is mildly associated with the development of HCA. Despite modern oral contraceptives containing lower doses of estrogen, this has been a new trend in the etiology of HCAs. Other risk factors include the use of anabolic-androgenic steroids; the use of other drugs including barbiturates, clomiphene, and recombinant human growth hormone; genetic syndromes including familial adenomatous polyposis and glycogen storage diseases; and environmental factors such as obesity and alcohol consumption [10]. The prevalence of obesity, the new major risk factor of HCA, is increasing worldwide. Obesity, hepatic steatosis, and metabolic syndrome are considered to be associated with the incidence of multiple HCAs [7]. The number of HCAs is not related to the incidence of complications such as bleeding or malignant transformation to HCC [10, 11]. Considering that our patient was a young man with severe obesity without any medication or drug use (including alcohol), obesity was the only risk factor for HCA development and may have been associated with the multiple lesions.

In the diagnosis of HCA, non-invasive tools such as imaging play an important role, in addition to biopsies. Among the imaging tools, CECT and MRI are useful for diagnosing HCA. HCA is typically hypervascular and heterogenous on the arterial phase but isoattenuating or hypoattenuating on the portal venous phase of CECT [9]. The diagnosis of HCA requires differentiation from other benign hepatic tumors, particularly focal nodular hyperplasia and HCC. MRI is considered the modality of choice in the differential diagnosis of HCA and its subtypes. Among the three major HCA subtypes, the β-catenin-activated subtype does not display specific imaging characteristics, but the findings may mimic those of HCC. Hepatocyte nuclear factor-1α (HNF-1α)-inactivated type HCA is typically hyperintense or isointense on T1-weighted imaging and isointense or slightly hyperintense on T2-weighted imaging; it demonstrates moderate arterial enhancement that does not persist into the portal venous phase on gadoxetic acid enhancement. Inflammatory type HCA is typically isointense or mildly hyperintense on T1-weighted imaging and diffusely hyperintense on T2-weighted imaging; it demonstrates intense arterial enhancement that persists into the portal venous and delayed phases on EOB-MRI [12]. The present case showed almost typical findings of inflammatory HCA. However, the largest tumor in segment 3/4 showed partial washout on the portal venous phase that was suggestive of HCC, thus requiring the percutaneous needle biopsy to exclude HCC.

According to the World Health Organization classification edited in 2010, HCA can be categorized into four subgroups: (i) HNF-1α-inactivated HCA (tumors with HNF-1α mutation with steatosis, no infiltrate, and negative liver fatty acid protein expression), (ii) β-catenin-activated HCA (tumors with β-catenin mutation with frequent cytological abnormalities), (iii) inflammatory HCA (telangiectatic/inflammatory HCA without HNF-1α or β-catenin activation, inflammatory infiltrates, and serum amyloid A-positive cells), and (iv) unclassified HCA (tumors without any mutation/activation and no inflammatory infiltrate) [13]. In our case, the pathological examination revealed sinusoidal dilatation and inflammatory cell invasion but no dysplasia. The immunohistochemical examination revealed that the tumor cells matched neither the HNF-1α-inactivated subtype (due to the presence of fatty acid-binding protein) nor the β-catenin-activated subtype (due to the absence of β-catenin and glutamine synthetase). Thus, the tumor was diagnosed as an inflammatory subtype with many positive cells for serum amyloid A protein. In the needle biopsy, however, the tumor was suspected as unclassified subtype because the cells tested were positive for serum amyloid A. However, no overexpression was noted. As such, this case was difficult to diagnose reliably using biopsy alone.

The management of HCA includes multidisciplinary treatments due to the nature of the associated complications that may include bleeding and malignant transformation. In a systematic review of 1176 patients with HCA, van Aalten et al. reported an overall frequency of 27.2% for hemorrhage and 17.5% for rupture and intraperitoneal bleeding [14]. The risk factors for hemorrhage include a diameter ≥ 35 mm, visualization of lesional arteries, localization in the left lateral liver, and exophytic growth [15]. In a systematic review of 1635 patients with HCA, Stoot et al. reported an overall frequency of 4.2% for malignant transformation [16]. Risk factors for malignant transformation include sex (male), tumor size, and β-catenin-activated subtype. Based on currently available clinical and molecular risk factors, a personalized treatment algorithm and indications for surgical management of HCA were proposed in a few previous reports [17–19]. According to those reports, surgical resection is generally recommended for patients at a substantial risk of complications, such as those with tumor size larger than 5 cm, a tumor increasing in size, presence of the β-catenin-activated subtype, imaging features suggestive of malignancy, concurrent dysplasia and/or the inability to rule out HCC, progressively rising α-fetoprotein levels, and male sex [17, 18]. Therefore, our patient was scheduled to undergo surgical resection due to the largest HCA, larger than 5 cm, and the associated potential risks of rupture and malignant transformation. Although there were no malignant findings in the specimen, a hemorrhage was noted, which had the possibility of rupturing during the natural course. Therefore, the surgical approach was considered appropriate to avoid any complications.

LLR is also considered a feasible option with comparable efficacy and safety relative to the open liver resection for HCAs. A multi-institutional study showed that laparoscopic surgery can reportedly achieve short-term outcomes similar to those of open surgery for HCAs and has the additional benefits of a reduced blood loss (93 vs. 196 mL, *p* < 0.001), a need for transfusion (8 vs. 24 red blood cell units, *p* < 0.001), and a shorter hospital stay (5 vs. 7 days, *p* < 0.001) [20]. LLR also has cosmetic benefits, especially in young women. However, LLR in obese patients is considered problematic. Despite this, several reports have stated that obesity should not be a contraindication for LLR [21, 22] and that LLR was less influenced by BMI and was beneficial in obese patients [23, 24]. In our case, despite the patient being severely obese (BMI 40 kg/m^2^), LLR of the three separate lesions was successfully performed without excessive blood loss.

Recent studies on obese female patients with HCA have reported successful treatment by weight loss or bariatric surgery [8, 9, 25]. These may be alternative options in managing HCA because of the effect of weight loss on HCA regression. However, Yamaguchi et al. reported the case of an obese patient with a 20-cm-diameter HCA who underwent transarterial embolization and right hepatectomy for tumor rupture despite a 20-kg weight loss [26]. They concluded that careful follow-up is necessary for these patients, even with successful weight loss, and that more radical treatment, including surgical resection and transarterial embolization, may be appropriate in obese patients with large telangiectatic HCAs to avoid the risk of a rupture. Additionally, it remains unclear whether weight loss is effective in male patients with HCA. Therefore, further studies are required to establish the indications for weight loss programs and bariatric surgery as optional treatment modalities. This is necessary as the current literature only reports on the successful management of HCA in small case series exclusively in women. In our case, surgical resection was considered appropriate because weight loss efforts using diet and exercise had failed and the tumor had not regressed. We concern that there is a possibility of recurrence unless the patient loses body weight, and as such are continuing to recommend weight loss as part of the postoperative management course.

## Conclusions

We reported a case of successful laparoscopic management of multiple HCAs in a young, obese, Japanese man. Our findings suggest that HCA should be considered in the differential diagnosis of liver tumors in the obese patients. Further studies are required to establish individualized HCA treatments in obese patients, in considering clinical and molecular risk factors.

## Data Availability

Not applicable.

## Abbreviations

BMI: Body mass index
CECT: Contrast-enhanced computed tomography
EOB-MRI: Gadoxetic acid-enhanced (EOB)-MRI
HCA: Hepatocellular adenoma
HCC: Hepatocellular carcinoma
LLR: Laparoscopic liver resection
PIVKA-II: Protein level induced by vitamin K absence or antagonist-II
